# Wireless Sensor Networks for Precision Agriculture: A Review of NPK Sensor Implementations

**DOI:** 10.3390/s24010051

**Published:** 2023-12-21

**Authors:** Purnawarman Musa, Herik Sugeru, Eri Prasetyo Wibowo

**Affiliations:** 1 Department of Electrical Engineering, Gunadarma University, Depok 16424, West Java, Indonesia; 2 Department of Agriculture Technology, Gunadarma University, Depok 16424, West Java, Indonesia; herik_sugeru@staff.gunadarma.ac.id; 3 Department of Information Technology, Gunadarma University, Depok 16424, West Java, Indonesia; eri@staff.gunadarma.ac.id

**Keywords:** macro–micro nutrients, nutrient sensor, nitrogen, phosphorus, potassium, soil nutrient assessment, crop

## Abstract

The integration of Wireless Sensor Networks (WSNs) into agricultural areas has had a significant impact and has provided new, more complex, efficient, and structured solutions for enhancing crop production. This study reviews the role of Wireless Sensor Networks (WSNs) in monitoring the macronutrient content of plants. This review study focuses on identifying the types of sensors used to measure macronutrients, determining sensor placement within agricultural areas, implementing wireless technology for sensor communication, and selecting device transmission intervals and ratings. The study of NPK (nitrogen, phosphorus, potassium) monitoring using sensor technology in precision agriculture is of high significance in efforts to improve agricultural productivity and efficiency. Incorporating Wireless Sensor Networks (WSNs) into the ongoing progress of proposed sensor node placement design has been a significant facet of this study. Meanwhile, the assessment based on soil samples analyzed for macronutrient content, conducted directly in relation to the comparison between the NPK sensors deployed in this research and the laboratory control sensors, reveals an error rate of 8.47% and can be deemed as a relatively satisfactory outcome. In addition to fostering technological innovations and precision farming solutions, in future this research aims to increase agricultural yields, particularly by enabling the cultivation of certain crops in locations different from their original ones.

## 1. Introduction

The substantial reduction in agricultural land can be attributed to various factors, including the relentless expansion of urban areas and the rapid industrialization that has transformed landscapes. This decline in available agricultural acreage has emerged as a complex and multifaceted issue, impacting food production and necessitating a deeper exploration of its underlying causes and consequences. Consequently, addressing this issue with modern technological solutions has become imperative. Moreover, the contemporary agricultural landscape presents a myriad of challenges, including but not limited to the pressing issue of water scarcity, the necessity for heightened fertilization practices, and the ever-shifting dynamics of climate patterns. These multifaceted challenges underscore the imperative to incorporate advanced technological solutions into our agricultural practices. This integration is essential not only for optimizing agricultural production but also for minimizing the wastage of precious resources.

Wireless Sensor Networks (WSNs) stand out as one of the leading technologies that have evolved in the field of agriculture. In the research conducted by the authors of [1,2], in their 2020 publication on soil monitoring, WSN are predominantly utilized to realize the fundamental conceptual framework of precision agriculture (PA). Precision agriculture represents a new paradigm in modern farming that combines information technology and communication to enhance land and resource management. It operates by observing phenomena using sensors and responding through actuators, thereby achieving the necessary parameter values and prerequisites for optimal crop health and yield production, even in the face of limited resources. In this study, the authors link the emergence of Wireless Sensor Network (WSN) technology as a potential solution for remote and real-time data monitoring and collection across agricultural fields.

The availability of nutrients holds significant importance in supporting plant growth and production. In the context of plant nutritional requirements, nutrients can be categorized into two primary groups, namely macronutrients and micronutrients. Among these macronutrients, nitrogen (N), phosphorus (P), and potassium (K), collectively referred to as NPK, play a pivotal role in providing essential nutritional support to plants. One crucial aspect of precision agriculture involves monitoring soil conditions, including the levels of nitrogen (N), phosphorus (P), and potassium (K), which are essential nutrients for plant growth. Effective nutrient management, particularly NPK, is of paramount importance in improving crop yields while minimizing environmental impact. Insufficient presence of these nutrients can lead to adverse effects on plants, such as reduced productivity, yellowing of leaves, and a decline in fruit quality, ultimately resulting in crop failure.

The research by the authors of [3] focuses on the development and application of Internet of Things (IoT)-based systems to assess soil nutrient content in the context of horticultural agriculture. They have developed the use of IoT sensors to measure the levels of nitrogen (N), phosphorus (P), potassium (K), and other nutrients in the soil with the aim of enhancing fertilizer management and crop yields in horticultural farming. The requirement to monitor soil nutrient levels is essential for the effective utilization of fertilizers and the mitigation of the ecological footprint resulting from fertilization techniques. Nevertheless, traditional soil assessment procedures, involving field soil sampling coupled with subsequent chemical analysis in a laboratory setting, are associated with significant expenditures and prolonged timelines [4]. The investigation conducted by the authors of [5] aimed to evaluate the potential correlation between the chemical constituents found in potato petioles and the spectral characteristics of the leaves. Additionally, the study sought to determine whether there exists a variance in correlation values when considering the spectral data of freshly harvested leaves as opposed to those that have been dried.

This research employed a systematic literature review approach with the objective of collecting and analyzing literature pertaining to the incorporation of NPK sensors within Wireless Sensor Networks (WSNs) for precision agriculture. Additionally, various other sensors, including those monitoring parameters like temperature, humidity, wind speed, solar radiation, and rainfall, play a supporting role in precision agriculture practices. The anticipated outcome of this study is to introduce innovations in the implementation of NPK sensors within WSNs, thereby opening new avenues for optimizing fertilizer utilization, averting nutrient imbalances in crops, and ultimately boosting crop yields.

## 2. Layout of Sensor Networks

The arrangement of sensor placements refers to how the location of a sensor is organized. When determining sensor placement, it should not be equated based on topology. Placement, which is at times denoted as the physical topology, holds a critical role within the context of sensor networks. Topology, in this context, signifies the arrangement of nodes that serves efficiency [6,7] and represents the pathways of information transmission. Placement, on the other hand, pertains to the tangible positioning of sensor nodes within the physical environment. The meticulous consideration of sensor placement stands as a matter of paramount importance.

In agricultural settings, the environment exhibits a remarkable level of dynamism, characterized by the spatial and temporal variations in various parameters. Over time, plants grow and, in doing so, exert an influence on the performance of sensors. To illustrate, take the example of a substantial greenhouse structure; within it, one may discern multiple micronutrient zones, each manifesting as a heterogeneous area with its own unique set of parameters. This, in turn, creates an environment that differs markedly from its surrounding zones.

To effectively monitor fluctuations in nutrient levels within this intricate agricultural landscape, it becomes imperative to deploy sensors non-uniformly. The establishment of suitable sensor placement locations can be guided by the patterns of irrigation and fertilization. Within the purview of this study, sensor placement is systematically categorized into horizontal and vertical layouts to enhance precision and effectiveness.

### 2.1. Horizontal Layout

The layout of conventional system sensors takes on a random or grid pattern [8]. Grid patterns typically require a minimum of six rows and six columns of intersecting nodes. The resulting grid can cover an area ranging from 20 to 50 m of farmland. Bridge nodes are placed along the outer edges of the agricultural area. This topology model can be used to cover larger areas, such as 30 × 30, using 900 sensor nodes placed at each intersection [9]. This allows for the design of a sensor layout to monitor the nutrition of a 6 × 9-m agricultural plot.

In another scenario, instead of placing sensor nodes in a grid layout, the authors propose dividing the geographical field area into grids and siting two–three nodes in each grid. In the context of greenhouse monitoring systems, it is a common practice to position nodes along the edges of a grid and these nodes are typically shared with neighboring grids. This arrangement is often supplemented by the placement of base stations at one end of the greenhouse, as observed in the study conducted by the authors of [10,11]. The advantage of this configuration is that nodes situated within the grid structure offer enhanced flexibility and provide more comprehensive coverage of unoccupied areas in comparison to configurations where nodes are placed at the intersections of the grid.

Furthermore, an alternative grid arrangement known as tessellation, as discussed in the study [12], presents a unique perspective. While traditional grids are often visualized as repetitive square or rectangular patterns, tessellation introduces a more intricate geometry. In tessellation, the grid is composed of tiles formed by regular polygons, which can take the form of triangles, squares, hexagons, and various other shapes. This diversity in polygonal shapes provides researchers with additional options for configuring the layout of nodes within the greenhouse, catering to specific monitoring requirements and spatial constraints.

**Figure 1:**
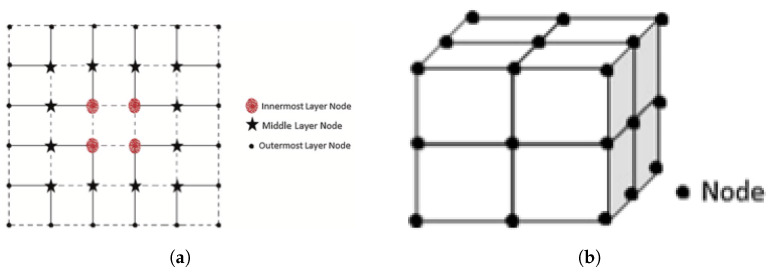
Layout for node sensor’s model and representation layers (source: [14]). (**a**) Legend of the layers of tessellations. (**b**) Hybrid layout.

Tessellation, a geometric concept, retains the fundamental attributes of grid structures while offering an added benefit of efficiently filling vacant areas within a defined space. This tessellation approach effectively circumvents issues related to overlap, ensuring a cohesive and unambiguous means of communication. Notably, nodes strategically positioned along the tessellation boundaries exhibit equidistant characteristics [13]. Building upon this foundational concept, the authors of the study extend their exploration by introducing the innovative notion of tessellation layers. In various instances of tessellations being visually depicted, Figure 1a provides an illustrative representation of the layers within these tessellations. Distinct notation is employed to denote nodes located within different layers. It is worth noting that these layers envelop the central point of the tessellation, resulting in a hierarchical arrangement. The mathematical connection between the quantity of nodes (*N*) and the quantification of stratification levels (*C*) is mathematically formulated as per Equation (1).

(1) $$N={\left(2C+1\right)}^{2},$$

Within the framework of the grid network architecture, a sophisticated hierarchical clustering topology is introduced. In this topology, parent nodes are strategically equipped with redundant nodes, strategically positioned to optimize network longevity. The innovative algorithm introduced in this research is officially referred to as the Redundant Node Deployment Algorithm (RNDA). RNDA effectively leverages the foundational principle underpinning the equitable allocation of external forces and stresses across structural components and support systems as a pivotal element in its operation with the aim of augmenting the network’s overall lifespan significantly. Remarkably, even with a minimal number of strategically placed redundant nodes, this approach has been shown to extend the network’s operational longevity to an impressive magnitude, encompassing thousands of rounds. In addition to the grid, random layouts are also straightforward, while the grid layout is the most commonly used [15], preferring to use directional antennas to construct a row layout. Transmitters are placed in front of each row to develop a path loss model.

During the initial phase dedicated to designing an optimal layout, it is imperative to strategically deploy sensors across the agricultural field to capture data on a range of external environmental parameters such as temperature, humidity, and rainfall, among others. The placement of external sensors can be considered as forming an autonomous topology, isolating them from the internal sensor nodes. In cases where inter-sensor communication is necessitated, it should be confined to their localized sphere of influence.

In order to investigate the influence of various factors on leaf growth, four specific environmental conditions were examined: well-ventilated, hot and low-humidity, humid, and warm areas [16]. Despite the uniform deployment of wireless sensors and cameras in our study, we took into consideration the diversity and dynamics of these regions when determining the density of sensor nodes in each area. To ensure a robust monitoring system within the greenhouse, in addition to stationary sensors, we also employed mobile sensors. A total of 120 sensor nodes and 4 gateway nodes were strategically positioned throughout the orchid greenhouse for monitoring purposes. Among these 120 sensor nodes, 52 remained stationary, while the remaining 68 moved at a speed of 0.15 m/s. We used the Dynamic Convergecast Tree Algorithm (DCTA) to reconfigure the network topology every 30 min. The furthest measured distance between sensor nodes was found to be 75.6 m [17].

In tomato plants, sensors are evenly distributed in a tree-cluster configuration, with routing nodes forming a triangular grid. In the realm of sensor networks and routing nodes, their interdependence is a notable factor, as established [18]. Beyond this interdependence, a more nuanced approach can be considered, involving the creation of discrete networks tailored to specific sensor types. A relevant example can be drawn from the work of the authors of [19], who proposed the segmentation of networks into dedicated clusters for soil sensor nodes and environmental sensor nodes. Furthermore, in the context of monitoring water quality for irrigation purposes, the deployment of a dedicated, isolated network becomes imperative. The concept of deploying distinct, isolated networks for soil and environmental sensors introduces a novel dimension to the realm of precision agriculture monitoring, potentially leading to enhanced precision and efficiency in data collection and analysis.

The strategic deployment of sensors within the layout, considering factors such as the quantity of sensors employed, their spatial distribution, and the specific parameters they measure, plays a pivotal role in determining overall system performance and data accuracy.

Scientists have employed a horizontal configuration of Wireless Sensor Networks (WSNs) to monitor and record a comprehensive array of environmental variables, encompassing air temperature, temperature, humidity, CO${}_{2}$ levels, light intensity, Vapor Pressure Deficit (VPD), soil moisture content, sap flow rate, stem diameter, and leaf thickness, as well as leaf wetness.

The scope of parameter variability encompasses distances as short as a few meters, encompassing factors such as soil temperature, soil moisture content, and soil pH levels, among others. While adopting a grid layout may seem like a viable option, it necessitates a greater number of sensors, consequently escalating the overall cost of implementation.

**Figure 2:**
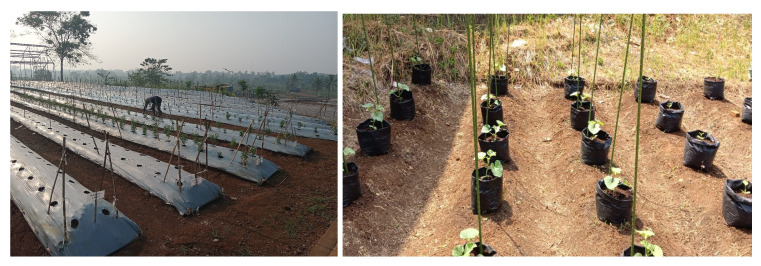
Our proposed research focuses on the horizontal layout.

In the realm of precision agriculture, monitoring activities encompass vast expanses of agricultural land. In this context, the integration of mobile nodes into the monitoring infrastructure can offer valuable auxiliary support. Particularly when dealing with parameters characterized by a limited range of variability, the deployment of mobile nodes emerges as a pragmatic solution to circumvent the potential pitfalls of network congestion and its associated operational costs. In the realm of touch-based sensors such as chlorophyll content meters, the integration of mobile robotic nodes can greatly enhance the efficiency and effectiveness of the monitoring process. The deployment of these nodes introduces the concept of isolating sensor and router network topologies, which serves to not only extend the lifespan of the network but also effectively manage and mitigate potential risks and challenges associated with data collection and transmission. In the context of a scientific manuscript with a more detailed and complex structure, we will conduct the following research:

In our research endeavor, several NPK sensor nodes will be strategically deployed across the agricultural field, symbolically representing the horizontal layout within the technological park of Gunadarma University, located in the village of Cianjur, within the province of West Java, as visually depicted in Figure 2.

### 2.2. Vertical Layout

In the initial stages of the sensor era, during a period when the cost of sensors was comparatively high for the purpose of greenhouse monitoring, the conventional practice for sensor deployment typically entailed the placement of a solitary sensor node positioned at the central point within the uppermost section. In accordance with the prognostications posited by Moore’s Law, the continual escalation in integration levels has played a pivotal role in the substantial reduction of costs. This significant cost reduction, in turn, has facilitated the widespread deployment of numerous sensors aimed at augmenting the precision and dependability of monitoring systems. Interestingly, certain propositions within the research community have even suggested the placement of all sensor nodes at a uniform elevation to further enhance system coherence and performance, as previously explored in studies such as Akkas et al.’s “IoT-Based Sensing and Monitoring” (2017) and Li’s investigation into the “Line-of-Sight Sensor Network” (2015) [20,21]. The growth and leafage of plants significantly affect the sensor communication range [18], making the vertical layout appear to be a prominent solution. The vertical farming layout model has seen widespread application, predominantly observed in urban residential settings, with a predominant focus on maximizing space utilization, often through the utilization of limited available space, even to the extent of attaching these structures to the walls of residential buildings.

The sensors are installed in a vertical layout because they monitor all plants’ growth in the vertical direction, either upwards or downwards. The farming model in paddy fields or open fields is referred to as terracing. Meanwhile, the vertical layout model has specific characteristics for climate control and monitoring in greenhouses. On the other hand, other authors suggested placing sensors at separate height levels [15,22,23,24,25]. Pahuja and colleagues used this model to monitor parameters at the canopy level and above the crop canopy [22]. Research by Harris and colleagues used the monitoring of several parameters for precision agriculture calibration. In previous research investigations, within an alternative adaptation of the vertical layout model, sensors were strategically placed within the soil, with only the coordinator placed at a higher level or in the middle in the case of a single coordinator [8,23,26].

Previous research proposed monitoring soil parameters, where sensors needed to be placed vertically below the ground [27]. Yu and his research team proposed a strategy in their study that involves the placement of the antenna at various elevations while maintaining the sensors at ground level [28]. This strategic approach was found to have a significant impact on improving the communication range within the agricultural environment. The outcomes indicated a direct correlation between the height of the antenna and the extent of communication coverage. However, this correlation plateaued at a height of 1 m since, at greater elevations, the presence of tomato plants no longer posed interference to the communication system. Consequently, the optimal height of the sensors is contingent upon the actual height of the plants being monitored.

Moreover, this vertical configuration exhibits substantial potential for facilitating the collection of critical plant growth-related parameters, such as the Normalized Difference Vegetation Index (*NDVI*). *NDVI* relies on the spectral data reflected from the crop canopy and can be mathematically represented by Equation (2). This methodological approach holds promise for more comprehensive and nuanced assessments of plant development and health in agricultural settings.

(2) $$NDVI=\frac{R_{ni}-R_{v}}{R_{ni}+R_{v}}$$

in which *R*${}_{ni}$ represents the near-infrared spectral reflectance of plants and *R*${}_{v}$ represents the spectral reflectance of visible light from plants.

In a manner analogous to the horizontal layout, the vertical configuration is contingent upon the specific parameters being measured. In the case of assessing soil chloride concentration or soil pH levels, it is imperative to orient the sensors in a vertically downward position. This positioning facilitates accurate data acquisition by ensuring direct contact and penetration into the soil medium, thus enabling precise analysis of the targeted soil properties. In the realm of environmental monitoring, the positioning of sensors plays a pivotal role in accurately capturing various parameters. Take, for instance, the measurement of wind speed; it necessitates the strategic placement of sensors in an outdoor setting, with due consideration to the minimum height above ground level. Similarly, the deployment of CO${}_{2}$ sensors requires a nuanced approach due to the inherent characteristic of carbon dioxide being denser than air. Consequently, to optimize CO${}_{2}$ monitoring, sensors are most effective when positioned beneath the plant canopy level.

Furthermore, when observing parameters like illumination or the light incident upon plant leaves, the placement of light sensors assumes significance. To mitigate the formation of shadow zones, it is imperative to position these sensors above the leaf surface, thereby ensuring a comprehensive assessment of light conditions.

Height level, as another critical factor, demands meticulous attention during sensor deployment. When situating sensors at various height zones, it becomes essential to account for the presence of the plant canopy. This consideration prevents unwanted interference in data collection. For example, in the context of monitoring tomato plants, the placement of node antennas should exceed 1 m above ground level to maintain consistent connectivity.

In instances where plant height undergoes significant variations throughout the growth cycle, as is often the case with pepper plants, a dynamic approach to node height is required. This may involve the periodic adjustment of sensor heights as the plants grow or the implementation of alternative solutions, such as the utilization of long-distance routing nodes. A comprehensive review of these principles can be found in the work [18].

This diverse range of configurations indicates the significance of the vertical layout in WSN applications and underscores the need for further investigation to optimize the deployment of sensors at varying heights. The choice of the number of nodes depends on the specific requirements and objectives of the environmental monitoring system under consideration. These findings collectively contribute to the body of knowledge regarding WSN deployments in environmental monitoring scenarios. In the context of agricultural monitoring, a comprehensive range of parameters is employed for data acquisition, including but not limited to temperature, humidity, carbon dioxide (CO${}_{2}$) levels, relative humidity, wind speed, meteorological conditions, and irradiance. These parameters play a pivotal role in ensuring precise and efficient agricultural management practices.

### 2.3. Hybrid Layout

In addition to the conventional horizontal and vertical sensor layouts, an innovative hybrid sensor layout has emerged as a potential solution. In a recent experimental study conducted by the authors of [17], depicted in Figure 1b, a novel 3D row–column–height grid structure for sensor node placement was introduced [29]. Both the traditional and the proposed hybrid layouts have demonstrated remarkable effectiveness in the context of plant monitoring, regardless of whether it is conducted within controlled environments such as greenhouses or in outdoor agricultural settings. Furthermore, Aiello and his research team proposed a comprehensive deployment strategy involving the placement of 20 sensor nodes distributed across five distinct locations and four varying heights, ranging from 0.7 m to 3.8 m above the ground surface [30].

Changes in one parameter can influence other parameters. The illumination or radiation intensity can affect temperature and humidity measurements. Ferentinos and colleagues examined the impact of various levels of radiation intensity on temperature and relative humidity measurement errors [31]. Hence, it is advisable to consider the positioning of sensor nodes, opting for either exposed configurations or partially enclosed setups, as empirical evidence suggests that exposed nodes tend to outperform fully enclosed ones. An alternative approach, as proposed by Kuroda and his research team [32], involves housing the nodes within protective enclosures. Additionally, the deployment of sensor nodes can follow hierarchical or master–slave architectures to optimize data collection and processing. In a tiered layout, specific sensor nodes are dedicated solely to data sensing, while others possess advanced capabilities for data collection and processing. Lower-tier nodes are responsible for transmitting measured data to higher-tier nodes, which can further process the data as needed or transmit it directly to the top-tier nodes. These upper-tier nodes are typically interfaced with a gateway or central repository, as discussed in the studies conducted [33,34].

## 3. Data Transmission Methods

Sensors need a method for transmitting data to the control center or users in need of information, as this is the fundamental function of sensors in WSNs. Sensor node communication can be wireless or a combination of wireless and wired. Various technologies can be utilized, as demonstrated in Table 1, where we provide a comparison of communication technologies based on parameters including range, frequency, network scalability, cost-effectiveness, data transmission rate, power efficiency, and communication modalities.

In Table 1, it is evident that each technology exhibits the capability to convey sampled data from sensors to the central control unit for subsequent analysis. Communication technologies characterized by high data transmission rates, such as WiFi and Bluetooth devices, are associated with elevated power consumption when compared to Zigbee. Another noteworthy observation pertains to the inherent trade-off between power consumption and device lifespan; devices with higher power consumption are naturally associated with shorter operational lifespans. Therefore, Zigbee emerges as a pragmatic choice for communication. Zigbee, Xstream, and LoRa all share the common attributes of offering extensive coverage with low data transmission rates within the peer-to-peer network framework. However, additional considerations encompass the prohibitively high cost associated with LoRa. Moreover, the LoRa platform is associated with significant latency due to the proliferation of LoRa devices. Conversely, while Xstream presents an economical option, its operational intricacies stem from the presence of numerous channels.

## 4. Monitoring Nutrition with NPK Sensors

According to [35], the nutritional requirements of a plant depend on the plant’s type, and the quantity of fertilizer to be used also relies on the existing NPK nutrient content in the soil. Ref. [36] also reports that the additional fertilizer required is influenced by the values of NPK present in the soil. Determining the range of NPK content in the soil becomes a crucial factor in optimizing fertilizer application (Table 2).

Fertilization that is effective must involve the precise selection of suitable fertilizer types, determination of the correct dosage, adherence to appropriate timing, and the implementation of the correct method of application. The excessive or inadequate application of fertilizers can lead to reduced production yields and relatively lower quality. In Table 3 presented below are fertilization recommendations that can be applied to various types of horticultural crops:

## 5. Results

### 5.1. Wireless Sensor Networks in Precision Agriculture

WSNs consist of spatially distributed sensor nodes that autonomously collect and transmit data to a central node. This network provides real-time monitoring capabilities, enabling farmers to make decisions based on current field conditions. In precision agriculture, WSNs are employed for various purposes, such as climate monitoring, soil moisture assessment, pest detection, and nutrient management.

Our study proposes the implementation of a Wireless Sensor Network (WSN) system for monitoring plant nutrition in the agricultural fields within the Technopark of Gunadarma University (UG-TP), situated in the village of Mande, Cianjur, West Java. The application of WSN in our research proposal utilizes a communication medium through a LoRa WAN transmission system, commencing from the NPK sensor nodes, routing mechanisms, and LAN coordinators in each zone, to data communication with the central control and monitoring station, also located within the UG-TP premises.

Furthermore, the arrangement of the WSN layout in this study is tailored to suit the agricultural area’s topographical conditions, which include sloping terrain. Thus, we suggest the implementation of a hybrid horizontal layout for the sensor nodes to accommodate these specific landscape features.

**Figure 3:**
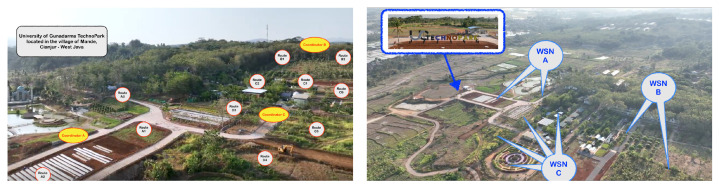
Our planning of a WSN system for coordination and routing.

In Figure 3, we have established the partitioning of the agricultural areas into three distinct WSN zones: WSN A for leafy vegetable crops, WSN B for leguminous plants, and WSN C dedicated to ornamental flora and various horticultural plants. Each WSN zone is equipped with a dedicated routing system, where each routing path accommodates a network of three to seven NPK sensor nodes.

In the context of inter-node sensor performance, the configuration of sensor node layouts for the three deployment scenarios can be tailored to specific requirements. However, based on the findings of our research analysis concerning the comparative evaluation of Wireless Sensor Network (WSN) layouts— horizontal, vertical, and hybrid—for agricultural applications, it is evident that implementing a vertical layout necessitates a significant number of sensor nodes, resulting in a considerable cost implication. Furthermore, this deployment approach also presents challenges related to the proximity of sensor nodes.

In contrast, for the horizontal and hybrid layouts, node sensor placements are characterized by a more balanced spatial distribution, avoiding excessively short inter-sensor node distances. In these configurations, increasing the number of sensor nodes can lead to the collection of more optimal information.

Precise farming is recommended for crops that require specific nutrient conditions within the soil. One notable aspect of our study is that our system can collect a wide range of data, encompassing NPK values, electrical conductivity (EC), temperature, soil moisture levels, and pH. This comprehensive data collection process introduces an innovative dimension to precision agriculture, where some crops cannot be grown in soil nutrient conditions that are less than ideal for the particular crop. Furthermore, the efficiency of the NPK sensors is underscored by their minimal energy consumption, while concurrently offering cost-saving benefits to farmers, thereby optimizing operational expenses.

### 5.2. Nutrient Management and NPK Sensor Monitoring

Optimal nutrient management is crucial for plant growth and health. Nitrogen, phosphorus, and potassium are primary nutrients that significantly impact plant development and crop yields. The integration of NPK sensors into WSNs allows continuous monitoring of nutrient levels in the soil, providing insights into plant nutrient requirements.

In the context of this scientific study, the soil measurement area employing NPK sensors represents four distinct geographical locations within the agricultural environment of the University Gunadarma Technopark.

### 5.3. Implementation of NPK Sensors in WSNs

Several technologies are utilized for NPK detection, including electrochemical, optical, and spectroscopic methods. These sensors measure various parameters such as electrical conductivity, pH, and nutrient concentrations in the soil. Integrating NPK sensors into WSNs involves addressing challenges like power consumption, data accuracy, and communication protocols.

Based on the macronutrient experimentation conducted, the results of the soil sample measurements obtained from the four specific soil sampling locations, as depicted in Table 4, reveal variations in nutrient concentrations among these distinct geographical sites. In this study, the evaluation of soil nutrient levels using the prototype designed within the scope of this research was juxtaposed with laboratory-grade instrumentation. The comparison yielded a valid outcome, indicating that the margin of error, ascertained through this analysis, averaged at 8.47%. The comparative results demonstrate satisfactory outcomes; however, the experimental data extracted from soil nutrient samples, encompassing NPK values, as well as parameters such as electrical conductivity (EC), pH levels, moisture content, and soil temperature, suggest suboptimal conditions for optimal crop growth. This necessitates further in-depth investigation by researchers to determine crop suitability and tailored recommendations based on the specific NPK composition and ancillary soil parameters for each distinct soil type.

### 5.4. Benefits and Challenges

Integrating NPK sensors into WSNs offers several benefits, including efficient resource utilization, reduced labor costs, and improved decision-making. However, challenges such as sensor calibration, data synchronization, and network maintenance need to be addressed for successful implementation.

## 6. Discussion

A more accurate monitoring of soil macronutrients allows for the efficient use of fertilizers, representing a novel solution and breakthrough in mitigating the costs and adverse environmental impacts associated with excessive fertilization. The strategic selection and deployment of NPK sensors as a policy could introduce a new hope for innovation in nutrient monitoring in agriculture, albeit not without its challenges. One of these challenges includes the initial procurement costs of the sensors and the readiness and training of farmers in their utilization.

In the realm of agricultural innovation, farmers are equipped with the capacity to promptly identify nuanced soil nutrient imbalances, encompassing both excesses and deficiencies in key macronutrients such as nitrogen, phosphorus, and potassium (NPK). This heightened awareness is made possible through the deployment and utilization of advanced NPK sensors. The incorporation of NPK sensors has the potential to enhance crop quality, yielding better outcomes for farmers. Regular maintenance and calibration of the sensors are essential to ensure measurement accuracy. The utilization of NPK sensors in soil nutrient monitoring necessitates the integration of sensor data with existing farm management systems and the requisite technical support.

## 7. Conclusions

Several implementations of NPK sensors within Wireless Sensor Networks for precision agriculture have been documented. Typically, these sensors are integrated into a system that continuously measures soil nutrient content in real-time and transmits this measurement data to data collection stations through wireless networks. Commonly used NPK sensors in this context include ion-selective sensors, near-infrared spectroscopy (NIR) sensors, and soil impedance sensors. The success of these implementations depends on factors such as sensor accuracy, transmission range, energy efficiency, and data integration.

However, challenges such as sensor reliability, low power consumption, and interoperability need to be addressed to ensure the sustainability and effectiveness of the system.

Integrated NPK sensors within Wireless Sensor Networks (WSNs) play a pivotal role in optimizing nutrient management to enhance crop productivity and sustainability. This review underscores the significance of sustained research and development efforts to address various challenges and unlock the full potential of NPK sensor implementations in precision agriculture.

Our research findings shed light on the importance of deploying Wireless Sensor Networks in precision agriculture, with a specific focus on NPK sensor monitoring. The utilization of NPK sensors within WSNs has the potential to bring about a transformative impact on crop nutrient management. This impact, in turn, has the potential to enhance agricultural productivity and support endeavors toward more sustainable agriculture.

## Figures and Tables

**Table 1:** Data transmission technology on WSNs.

Transmission Modules and Range	Frequency and Data Rate	Node	Cost and Energy	Communication Type
Zigbee (10–20 m)	2.4 GHz (20–250 Kbps)	65,000 nodes per network	L and L	Peer to peer
GPRS (in range of mobile network area)	900–1800 MHz (56–114 Kbps)	1000 nodes per network	H and H	Base station to device
LoRa (>10 Km)	169 MHz, 868 MHz, and 433 MHz (0.3–50 Kbps)	10,000 nodes per gateway	M and L	Peer to peer
Bluetooth (1–100 m)	2.4–2.485 GHz (1–3 Mbps)	8 active nodes per piconet	L and M	Master–slave and peer to peer
WiFi (20–100 m)	2.4 GHz (2–54 Gbps)	32 nodes per network	H and H	Access point to device
Xstream (5–16 Km)	2.4 GHz (10–20 Kbps)	7 channels, 65,000/channels	L and L	Peer to peer

Source: [14]. Note: L (Low), M (Moderate), and H (High).

**Table 2:** NPK level and range.

Level	Range (kg/ha)		
	**Nitrogen**	**Phosphorus**	**Potassium**
Low	0–280	0–11	0–118
Medium	280–450	11–22	118–280
High	>450	>22	>280

Source: [1].

**Table 3:** The optimal NPK application rate for horticultural crops.

Crops	Recommended Dose of NPK (kg/ha)		
	Nitrogen (N)	Phosphorus (P${}_{2}$O${}_{5}$)	Potassium (K${}_{2}$O)
**Fruit Crops**			
Banana	620	310	620
Mango	75	20	70
Citrus	110	35	55
Papaya	925	925	925
Guava	250	175	175
Apple	320	320	320
Pineapple	275	70	200
Sapota	100	50	50
Grapes	300	300	600
Pomegranate	500	425	975
Litchi	50	50	25
**Vegetable Crops**			
Potato	60	100	120
Tomato	180	120	150
Onion	125	75	125
Brinjal	180	150	120
Tapioca	45	90	120
Cabbage	150	125	100
Cauliflower	150	100	100
Okra	100	50	50
Peas	25	75	60
Sweet Potato	20	40	60
Chilli	150	75	75
**Plantation Crops**			
Coconut	100	55	210
Cashewnut	100	40	60
Arecanut	140	55	200
Cocoa	70	30	100
**Spice Crops**			
Garlic	40	75	75
Turmeric	150	60	108
Ginger	37.5	50	37.5
Cumin	30	20	20
Coriander	10	40	20
Tamarind	20	15	25
Fenugreek	30	25	40
Fennel	50	10	10
Pepper	110	50	155
Cardamom	75	75	150
Ajwan	40	20	20
Nutmeg	187.5	187.5	600

Source: [37].

**Table 4:** The results of comparison of NPK base location.

Area Soil Sampling	N		P		K		pH		EC		Moisture		Temp.	
	**IL**	**OS**	**IL**	**OS**	**IL**	**OS**	**IL**	**OS**	**IL**	**OS**	**IL**	**OS**	**IL**	**OS**
A	43	27	70	110	145	103	6	5	469	318	48	52	28	
B	33	14	51	79	98	72	7	5	349	254	47	51	28	27
C	43	35	72	128	140	121	6	4	441	356	50	64	28	28
D	5	4	515	310	47	56	29	28	44	226	73	106	160	99
Average	31	20	177	157	108	88	12	10	326	289	55	68	61	45
Error	5.46%		10.13%		9.75%		0.69%		18.63%		6.88%		7.76%	

Note: IL (Instrumens Lab.) and OS (Our System).

## Data Availability

The data presented in this study are available on request from the corresponding author.
